# Thyroiditis process as a predictive factor of sternotomy in the treatment of cervico-mediastinal goiter

**DOI:** 10.1186/s12893-019-0474-z

**Published:** 2019-04-24

**Authors:** Claudio Casella, Sarah Molfino, Carlo Cappelli, Federica Salvoldi, Mauro Roberto Benvenuti, Nazario Portolani

**Affiliations:** 1 Department of Molecular and Translational Medicine, Spedali Civili, 3rd Division of General Surgery, University of Brescia, P.zle Spedali Civili 1, 25123 Brescia, Italy; 2 Department of Clinical and Experimental Sciences, Surgical Clinic, University of Brescia, Brescia, Italy; 3 Department of Clinical and Experimental Sciences, Endocrine and Metabolic Unit, Medical Clinic, University of Brescia, Brescia, Italy; 4 Department of Cardio-Thoracic Surgery, Spedali Civili, Brescia, Italy

**Keywords:** Thyroidectomy, Cervico-mediastinal goiter, Thyroiditis, Sternotomy

## Abstract

**Background:**

About 10% of cervico-mediastinal goiter need to associate cervicotomy with a total or partial sternotomy to allow a safe removal of the goiter.

Aim of this study is to identify preoperative predictors of sternotomy for mediastinal goiter.

**Methods:**

Between January 2008 and December 2015, 586 patients were submitted to total thyroidectomy at Surgical Clinic of Brescia, Italy.

Among these, patients with cervico-mediastinal goiter have been divided in two groups based on the necessity of an associated sternotomy in the operating field: Group 1 (*n* = 40 patients) did not need sternotomy and Group 2 (n = 4 patients) underwent cervicotomy associated with sternotomy.

Clinical and pathological characteristics of patients were retrospectivelly recorded.

**Results:**

Among study group, 44 patients had cervico-mediastinal goiter. Thoracic CT was performed in all patients: an extension above aortic arch was found in 41 patients (93.18%) while an extention below aortic arch was found in 3 patients (6.82%).

The extension of the goiter below the aortic arch resulted as a predictive value in the choice of surgical treatment (*p* = 0.0001).

The thyroiditis process was found to be a significant predictive of the extention to a sternotomic approach (*p* = 0.029).

The years of goiter’s presence were on average 8.40 years in Group 1 and 14.75 years in Group 2.

These parameters proved to be predictive when choosing a cervicotomy with sternotomy.

Conclusions: Our study, despite limitations posed by small sample and its retrospective analisys, highlights the role of goiter’s extention (below the aortic arch), disease length (for more than 14.75 years) and flogistic process (positivity of Tg Ab and anti-TPO-Ab) in the choice of combined (cervicotomic and sternotomic) approach to goiter’s removal.

## Background

The definitions of cervico-mediastinal goiter are various: the most widely used identifies it as a volumetric increase of the thyroid volume, below the upper thoracic thigh with neck in hyperesthesium, for at least two transverse fingers (i.e. 3 cm) [1].

The incidence of this disease varies from 2 to 25%, with an average of 7–8% [2–4].

There is consensus among researchers about the presence of this pathology as a condition that requires a surgical treatment [2, 5, 6].

Between 90 to 97% of cases a radical removal of cervico-mediastinal goiter is possible through cervicotomy [1, 7].

In 2–8% of cases it is necessary to associate cervicotomy with a total or partial sternotomy, to allow an extension of the operative field and a safe removal of the goiter [6, 8].

Several Authors have considered various parameters, related both to the clinical characteristics of the patient and to the characteristics of the cervico-mediastinal goiter, in order to plan the appropriate eradicating surgical approach [9, 17].

In particular in a previous publication [3] we have shown how the goiter’s extention below the aortic arch, its development in the posterior mediastinum and the presence of the goiter itself for over 160 months are predictive values for a cervicotomy followed by total or partial sternotomy.

The purpose of this study is to help identify additional predictive factors, such as the presence of a thyroiditis process and the total thyroid volume, in order to plan a tailored surgical approach.

## Methods

Between January 2008 and December 2015, 586 patients with goiter, submitted to total thyroidectomy surgery, were retrospective enrolled at Surgical Clinic of the University of Brescia, Italy.

Among the study group, 462 (78.84%) were female, 124 (21.16%) male, with an F:M ratio of 4:1.

For each patient, age, symptomatology, years of goiter presence and thyroid function tests were recorded, and anti-thyroglobulin antibodies (anti Tg Ab) and anti-peroxidase (anti TPO-Ab) antibodies were taken into account.

Data from preoperative instrumental examinations (standard chest and trachea X-ray, neck ultrasound, thyroid scintigraphy, and eventual needle biopsy) were reported for each patient.

Thoracic CT scan was performed when the standard examinations hinted a mediastinal goiter extension. The mean volume of each thyroid was also assessed from the data obtained with the CT.

The type of implemented surgical approach was then recorded by dividing the patients into 2 groups: those who underwent cervicotomy (Group 1) and those who underwent cervicotomy plus partial sternotomy (Group 2).

The outcomes of the anatomo-pathological examination, as well as morbidity and post-operative mortality, have been recorded.

### Statistical analysis

The statistical analysis first studied the continuous data distributions (ages, etc.) by Student’s *t* test and Gaussian distribution adaptability test.

All the variables resulted as non-parametric except age and calcification and therefore in addition to the mean, the median and maximum/minimum annexes were presented as a measure of the central tendency.

For inferential analysis, the results of Chi^2^ tests were tested for discrete values ​​in contingency tables and Fisher’s exact test analysis and for continuous variables U-Mann Whitney’s non-parametric test was used to test any significant differences at the level of α = 0.05, as well as a univariate and multivariate regressive logistic methodology.

Software used for processing: SPSS© version 23.

## Results

Based on CT data and in accordance with the definition used [1], mediastinal goiters were 44, with an incidence of 7.51%.

Clinical-pathological characteristics of goiter and patients of Group 1 – no sternotomy (*n* = 40 patients) and Group 2 – associated sternotomy (n = 4 patients) are summarized in Table 1.

**Table 1:** Clinical-pathological characteristics of goiter and patients of Group 1 – no sternotomy (*n* = 40 patients) and Group 2 – associated sternotomy (*n* = 4 patients)

		Group 1 (*n* = 40)	Group 2 (*n* = 4)	*p*
Age	years (medium ± SD)	58,63 ± 9,67	67,5 ± 4,36	0,075
	(range)	(39–79)	(64–73)	
fT4	ng/dL (medium ± SD)	9,45 ± 2,09	6,52 ± 3,78	0,068
	(range)	(2,0 - 14,3)	(0,9 - 9,0)	
TSH	mUI/L (medium ± SD)	1,45 ± 2,27	1,32 ± 0,73	0,515
	(range)	(0,073 - 13,62)	(0,278 - 1,97)	
Presence of goiter	years (medium ± SD)	8,40 ± 4,27	14,75 ± 1,89	0,008
	(range)	(3–20)	(12–16)	
Thyroid’s weight	gr (medium ± SD)	127,00 ± 73,18	227,00 ± 32,19	0,02
	(range)	(45–385)	(193–257)	
Post-operative serum calcium	mg/dL (medium ± SD)	8,39 ± 0,67	9,17 ± 0,84	0,169
	(range)	(7,4 - 10,7)	(8,2 - 9,7)	
PTH	pg/mL (medium ± SD)	18,74 ± 27,47	15,50 ± 12,38	0,875
	(range)	(1,3 - 140,0)	(1,5 - 25,0)	
Presence of symptoms	yes (%)	23 (57,5%)	3 (75,0%)	0,455
	not (%)	17 (42,5%)	1 (25,0%)	
Extension to the aortic arch	above (%)	40 (100%)	1 (25,0%)	0,0001
	below (%)	0 (0%)	3 (75,0%)	
Lateral extension of goiter	right (%)	11 (27,5%)	1 (25,0%)	0,583
	left (%)	25 (62,5%)	2 (50,0%)	
	bilateral (%)	4 (10,0%)	1 (25,0%)	
Histological malignancy	yes (%)	6 (15,0%)	1 (25,0%)	0,513
	not (%)	34 (85,0%)	3 (75,0%)	
Histological thyroiditis	yes (%)	6 (15,0%)	3 (75,0%)	0,023
	not (%)	34 (85,0%)	1 (25,0%)	

No significant difference was found regarding age between patients of Group 1 and 2.

The years of goiter’s presence were on average 8.40 years in Group 1 and 14.75 years in Group 2.

This parameter proved to be predictive when choosing a cervicotomy with sternotomy.

The frequency of symptoms reported by patients is shown in Table 2. Overall, 26 patients (59.09%) suffered from symptoms, including the most frequent dyspnea (50%). A tracheal deviation or compression was found in 100% of patients. The presence of symptoms was not a significant parameter for the type of surgical approach.

**Table 2:** Frequency of symptoms in Group 1 – no sternotomy (*n* = 40 patients) and Group 2 – associated sternotomy (*n* = 4 patients)

	Group 1	Group 2	Total
Asymptomatic	17	1	18
Dyspnea	19	3	22
Dysphonia	2	0	2
Dysphagia	10	2	12
Cough	1	0	1

## 28 (63.64%) patients were euthyroid, 2 (4.54%) hyperthyroid, 14 (31.82%) hypothyroid

Thyroid function was not predictive of the type of surgery.

Among laboratory analysis, 9 patients (20.45%) had elevated antibody levels (anti Tg Ab and anti TPO-Ab), indicating a thyroiditis process and in 3 cases of these patients (33.33%), a combined intervention (cervicotomy associated with sternotomy) was required.

The cytological examination by needle biopsy was carried out to 19 patients (43.2%), which highlighted the presence of a papillary carcinoma in 5 cases (26.32%).

Standard chest X-ray gave cervical-mediastinic goiter as a result in all 44 patients (100%).

Thoracic CT was performed in all 44 patients: an extension above aortic arch was found in 41 cases (93.18%) and below aortic arch in 3 patients (6.82%).

The extension of the goiter below the aortic arch appeared to be a predictive value in the choice of surgical treatment (*p* = 0.0001), Table 1.

In addition to extension to the aortic arch, the lateral extension of goiter was also evaluated: in 12 cases (27.27%) an extension towards the right side was recorded, in 27 (61.37%) an extension towards the left side and in 5 (11.36%) a bilateral extension was found. These parameters (Table 1) do not correlate with the type of surgery.

In 15 patients (34.09%) scintigraphy with TC-99 m was performed: in 1 (6.66%) patient there was a spread goiter, in 5 (33.33%) cases there was a multinodular goiter with hot nodules and in 9 (60%) patients a multinodular goiter with cold nodules was detected (Table 3).

**Table 3:** Histology of substernal goiters in Group 1 – no sternotomy (*n* = 40 patients) and Group 2 – associated sternotomy (*n* = 4 patients)

	Group 1	Group 2	Total
Multinodular	25	2	27
Thyroiditis	6	1	7
Carcinoma	6	1	7
Basedow	2	0	2
Plummer Adenoma	1	0	1

Histological examination showed benign disease in 37 (84.09%) patients, while a papillary carcinoma was detected in the other 7 (15.91%).

The presence of malignancy was not predictive of a combined intervention.

Final histological examination confirmed the presence of a thyroiditis process in the 9 patients who already had shown high antibody levels in the laboratory exams.

The thyroiditis process was significantly correlated with the need to perform a cervicotomy plus sternotomy with a significance (*p* = 0.029).

By evaluating the same parameter (antibodies) through a univariate logistic regression, a significance was highlighted, which then confirmed the above-mentioned test.

A multivariate regression with two independent covariates, volume of the gland and antibodies, was also performed, but no significant evidence was demonstrated for an associated surgical approach.

Therefore, the presence of antibodies against Tg Ab and anti TPO-Ab is predictive of a cervicotomic and sternotomic approach, while there is no evidence of a relation between volume-antibody presence and choice of surgical treatment.

The weight in grams after surgical removal was on average 133.97 g. Particularly in Group 1, the average value was 127 g, in Group 2 of 227 g. This parameter was predictive in the choice of cervicotomy associated with sternotomy.

A correlation between the weight of the removed thyroid and the presence or absence of a high antibody title was also studied: although there is a difference in weight among patients with thyroiditis (107 g for goiter in patients without thyroiditis versus 137 g for those with thyroiditis) statistical analysis did not yield significant results.

Postoperative morbidity was due to transient hypocalcemia, resolved within 10 days after surgery, in 10 cases (22.73%), 9 in Group 1 (90%), and 1 in Group 2 (10%).

Post-intervention hypocalcemia did not result significant in the two patient groups.

No patient experienced persistent hypoparathyroidism.

Under no circumstances there was transient or permanent laryngeal nerve lesion and there were no cases of mortality.

## Discussion

In Literature there are different definitions of substernal goiters, as well as different ways to evaluate the actual extension. The frequencies of this pathology reported by the various Authors with regard to epidemiology and symptomatology are various and so are the suggested surgical approaches [3–5, 18–21].

Between 90 and 97% of cases it is possible to remove a cervico-mediastinal goiter with cervicotomy only [2, 7].

An avarange from 2 to 8% of substernal goiters require removal through a combined approach with cervicotomy and partial or total sternotomy [8].

Sternotomy extends the operative field, facilitating dissection, decreasing the risk of recurrent nerve lesions and helping hemostasis in lesions of mediastinal goiter’s vessels [6] in case of occurrence.

Up today it is difficult to preoperatively identify the right surgical field extention and several Authors have highlighted how various factors may affect the choice of surgical treatment [22].

It has been underlined that the presence in the mediastinum of a high density thyroid tissue evaluated by TAC is a highly indicative factor for the execution of an associated sternotomy [23].

For Riffat et al. [24] the indication to sternotomy depends on a few factors: the extension of the goiter below the hull, documented at the preoperative TAC, than the posterior mediastinal extension, the presence of ectopic nodules and the conical shape of the goiter when it is compressed by a narrow thoracic higher isthmic.

According to Qureishi et al. [25] predictive factors of an associated surgical approach are: the extension under the aortic arch or in the posterior mediastinum, the handlebar form, the discrepancy between chest diameter and the one of the goiter.

Other Authors [26] state that a sternotomy is necessary when the goiter has an extension below the aortic arch with chest compression symptoms.

In our study a cervicotomy was performed in 40 (90.91%) patients, while the association with partial sternotomy (sternal split) became necessary in 4 (9.09%) patients. The need to perform a sternal split was due to the extension of the substernal goiter below the aortic arch, in 3 cases (75%), while in 1 case (25%) it was chosen because of a discrepancy between the thoracic diameter and the goiter’s one, which did not allow a safe removal of the gland.

In our current and prior experience [3, 6] people who needed the combined approach were sick for twice as long as subjects that were submitted to cervicotomy only: years of disease were 8.40 in Group 1 and 14.75 years in group 2.

We have already confirmed how the age of patients, the presence or absence of symptomatology and thyroid function are not related to the need of sternotomy.

In recent years, the concept of “difficult thyroidectomy” has been introduced: this term is intended to identify a number of factors (topographical, technical and anatomical) which, alone or in association, make thyroidectomy more complex [15].

The thyroiditis process is recognized as a possible cause of complex surgery [14].

As a matter of fact, among the various determinant criteria for the so-called “difficult thyroidectomy” some Authors, [14] include autoimmune pathologies, regardless of the thyroid’s degree of functioning, pointing out that this data is valuable also for the presence of relatively small and sclerotic thyroids. For this reason, cases of so-called “thyroiditis” are one of the contraindications for minimally invasive surgery.

Di Vincenzo et All. [27] state that a cervicotomic approach associated with sternotomy allows a safe resection of large thyroid masses in close proximity to mediastinal structures and it is also necessary in other cases, including the presence of thyroiditis.

Other Authors [28] also created a Difficulty Scale to identify predictors of difficult thyroidectomy. Among the factors that have been statistically associated with longer operating times and increased complications, there is the presence of anti-Tg Ab antibodies, therefore again in the context of thyroiditis.

The predictive factors for a “difficult thyroidectomy” and the consequent risks of surgical complications are well described in a large Italian multicentric series [29]. It is essential to predict, for example, the risk of a recurrent nerve injury, considering that it does not always depend on an error of the surgeon [30]. Similarly, it is essential to predict the risk of any additional surgical access, such as sternotomy.

In our data the presence of a thyroiditis process was significantly correlated (*p* = 0.029) with the need to associate cervicotomy with sternotomy.

Similar conclusions have been found by other Authors [31].

**Fig. 1:**
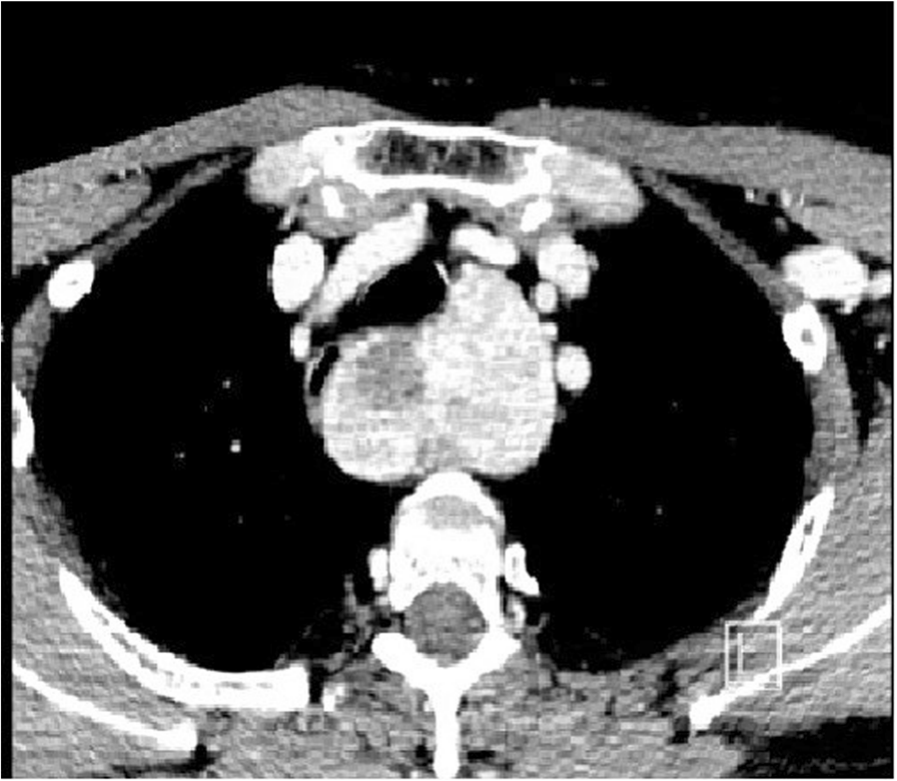
CT image of a substernal goiter that deepens beneath the aortic arch

As already mentioned in another work [3] the extension of the goiter under the aortic arch (Fig. 1), documented at the CT, was significantly correlated (*p* = 0,001) with a cervicotomy associated with partial sternotomy. This evidence has already been widely validated and described in Literature and it is now considered one of the main features for the choice of surgical treatment in substernal goiters [5, 6, 9, 10–13, 16, 32].

The extension of the goiter to the right, to the left or bilateral did not prove to be predictive in the choice of surgical treatment.

The gland’s volume did not result as a predictive parameter for the execution of an associated sternotomy approach in the sample tested. Indeed, this parameter, individually evaluated, is difficult to interpret, since, from a surgical point of view, after the binding of the cervical vessels at the beginning of the surgery, there is always a known atrophy of the gland that can allow it to be removed despite a significant volume at first.

The presence of a great volume associated with a high antibody title was also evaluated to see if there is a direct relationship between these two parameters and the type of surgical approach to be performed. The multivariate regression with two independent covariates x does not highlight in this sample an established link at a statistic level. On the other hand, this result is difficult to interpret because, despite the considered period, a rare intervention is being studied, and therefore the size of the sample is not wide.

The weight in grams of the gland after surgical removal resulted predictive in the choice of cervicotomy associated with sternotomy. However, this parameter cannot be considered a predictor since it is post-operative information.

The relationship between the weight of the thyroid and the presence or not of a flogistic process evaluated by antibody TG and anti-Tp Ab antibodies was also studied: although there is a weight difference (107 g for goiter in patients without thyroiditis versus 137 g for those with thyroiditis) between the two subgroups, this parameter was not statistically significant (*p* = 0.287).

Histological examination showed benign disease in 37 (84.09%) patients, while the remaining 7 (15.91%) diagnosis was of papillary carcinoma. The statistical analysis between the presence or absence of malignancy and the surgical approach was not predictive of the surgical choice.

## Conclusions

Our study, despite limitations posed by small sample and its retrospective analisys, highlights the role of goiter’s extention (below the aortic arch), disease length (for more than 14.75 years) and flogistic process (positivity of Tg Ab and anti-TPO-Ab) in the choice of combined (cervicotomic and sternotomic) approach to goiter’s removal.

## Abbreviations

Anti-TPO-Ab: Anti-peroxidase antibodies
CT: Computed tomography
TgAb: Anti-thyroglobulin antibodies
